# Medical and Legal Implications of MRI Scout Imaging in a Surgical Patient with Case Presentation

**DOI:** 10.7759/cureus.6833

**Published:** 2020-01-31

**Authors:** Stephen Albano, Ajay Ramnot, Javed Siddiqi, Deependra Mahato

**Affiliations:** 1 Neurosurgery, Desert Regional Medical Center, Palm Springs, USA

**Keywords:** angiolipoma, scout imaging, mri, spine surgery, tumor, medical and legal aspects

## Abstract

Spinal epidural angiolipoma is an uncommon finding; this case is presented to display the medical and legal implications of MRI scout imaging. In this case, a preceding period of ambiguous and non-focal symptoms led to an MRI of the lumbar spine without contrast with a scout image that captured a thoracic lesion. Review of the scout film led to a subsequent MRI of the thoracic spine with and without contrast that aided clinical decision making leading to surgical resection of the identified lesion and resolution of symptoms for this patient. The use of scout imaging has been described in the literature, but no concise agreement among physicians or professional medical societies exists regarding what utility, if any, may be obtained from the review of scout imaging. A discussion of medical legal implications of MRI scout imaging follows.

## Introduction

Scout images obtained during a computed tomography (CT) or magnetic resonance imaging (MRI) are used for localization purposes. The scout images are a survey of the region of interest used by the technician to select the area of dedicated image acquisition. While the scout images are typically not used for diagnostic purposes, some lesions can be identified on the scout images that are not on the dedicated sequences obtained later. In a study by Sener et al., 31 of 122 patients had a pathologic finding on scout which was not identified in the CT sequence [1]. These 25% of pathologic findings may not be identified since scout views are only intermittently evaluated by radiologists with no clear guidelines on whether physicians can be found negligent for not interpreting the images [2]. In some cases, start and end points of a scan are set by reference mark and a CT scout might not be obtained [3]. A case presentation follows to demonstrate the utility of MRI scout imaging, and discusses the medical and legal implications of interpreting vs disregarding scout imaging.

## Case presentation

**Figure 1:**
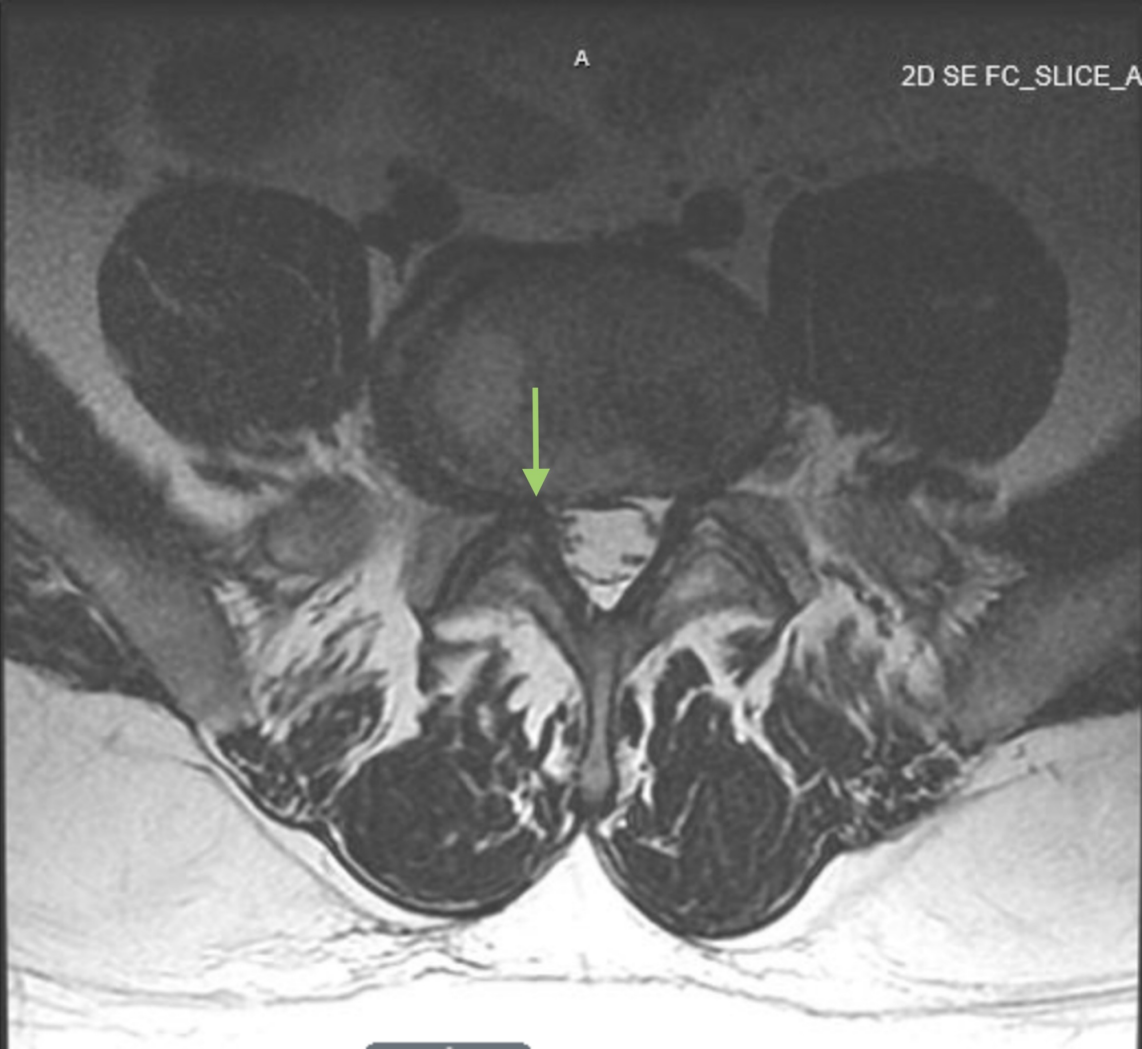
MRI lumbar spine T2-weighted axial image. Arrow showing L5-S1 disc herniation causing narrowing of the right neural foramen and impingement of the exiting L5 nerve root.

A 31-year-old Caucasian male with a history of lumbar disc herniations identified on an MRI of the lumbar spine one year ago presented to the emergency department (ED) for radicular back pain to the right lower extremity. Three weeks prior to presentation, the patient went on a jet ski trip where he incurred repetitive axial loading forces. Since that time, the patient noted increasing back pain with radiation to right lower extremity including the foot. The pain was intermittent and diffuse without a clear dermatomal distribution. The pain was described as electric, exacerbated with extension of his back, and relieved with flexion. No loss of bowel or bladder control was reported. On exam, the patient was 5/5 in the bilateral upper and lower extremities. Rectal tone and bulbocavernosus reflexes were intact. The patient underwent an MRI of the lumbar spine without contrast demonstrating a herniated L5-S1 disc as shown in Figure 1. The patient was prescribed a Medrol dosepak and gabapentin with instructions to follow up in clinic with plans for outpatient conservative management.

**Figure 2:**
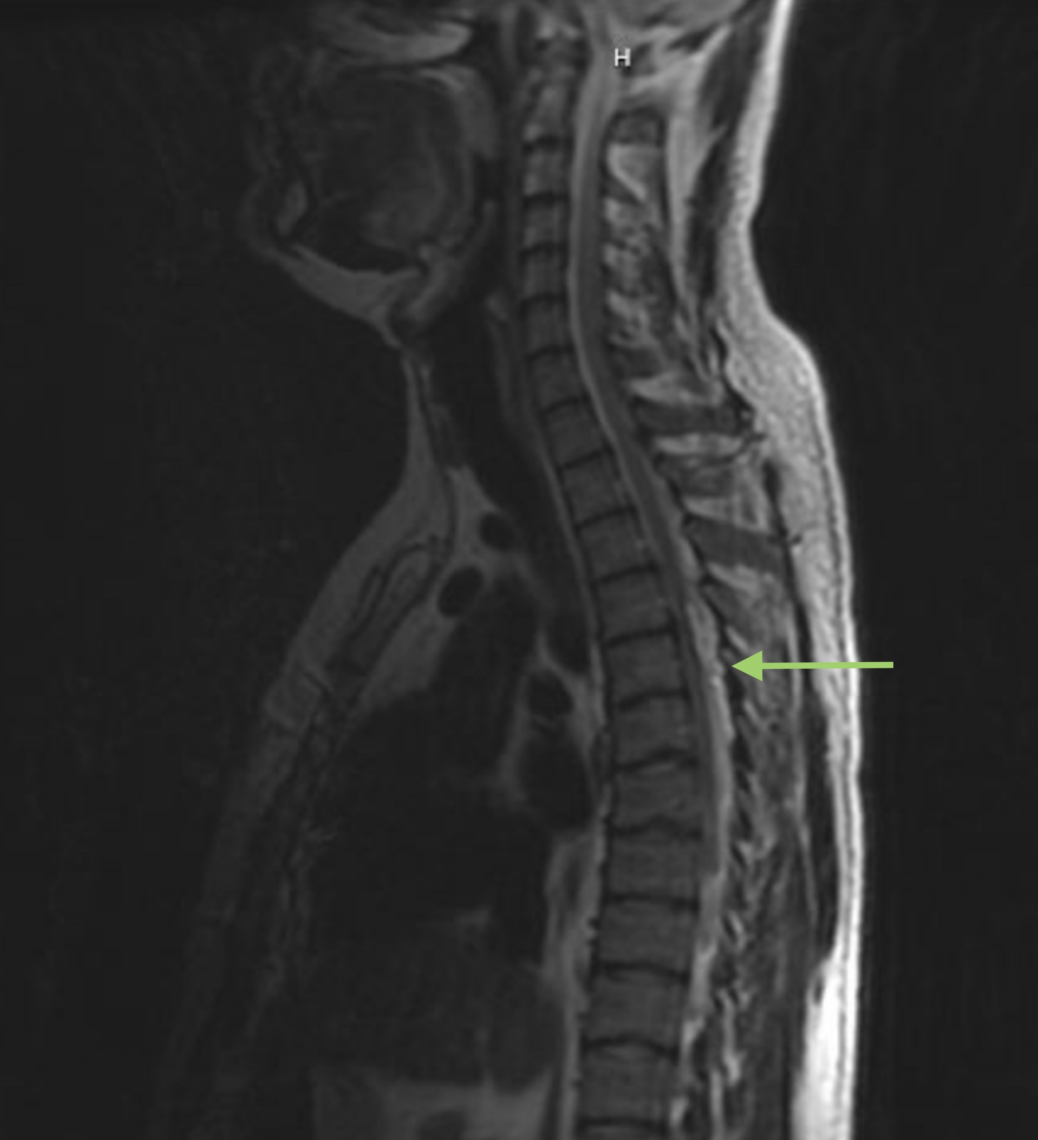
Sagittal MRI lumbar spine scout. Scout imaging revealed a hyperintense extradural lesion at the T4-T5 level indicated by the arrow.

**Figure 3:**
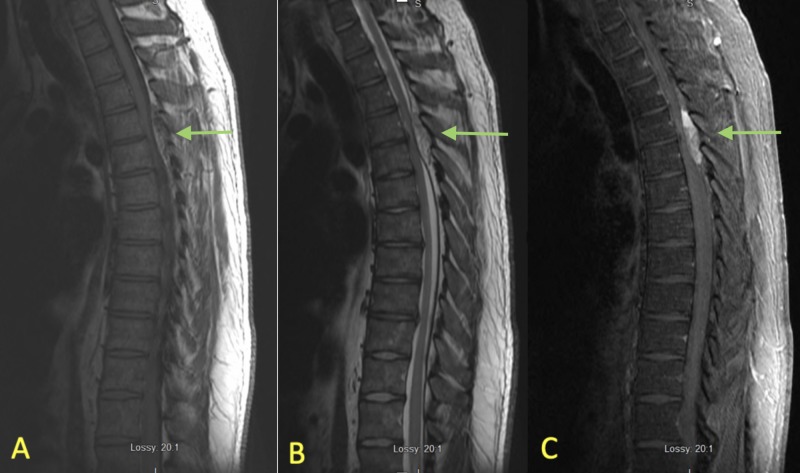
MRI thoracic spine sagittal T1 (A), T2 (B), T1 fat suppression with contrast (C). Panel (A) demonstrates a mixed intensity lesion at the T4-T5 region seen on T1 sequence indicated by the arrow. Panel (B) demonstrates mixed intensity on T2 sequence indicated by the arrow. Panel (C) demonstrates a contrast enhancing lesion indicated by the arrow.

At follow-up clinic visit, review of the MRI scout image included in the lumbar spine series demonstrated a thoracic abnormality as shown in Figure 2. MRI of the thoracic spine with and without contrast was ordered. It demonstrated T4-5 epidural mass as shown in Figure 3.

The patient was contacted and advised to go to the ED for evaluation. After examination, the images were reviewed with the patient. After a discussion of alternatives and the patient’s slowly progressing symptoms with an unidentified mass, the recommendation was for surgical resection.

**Figure 4:**
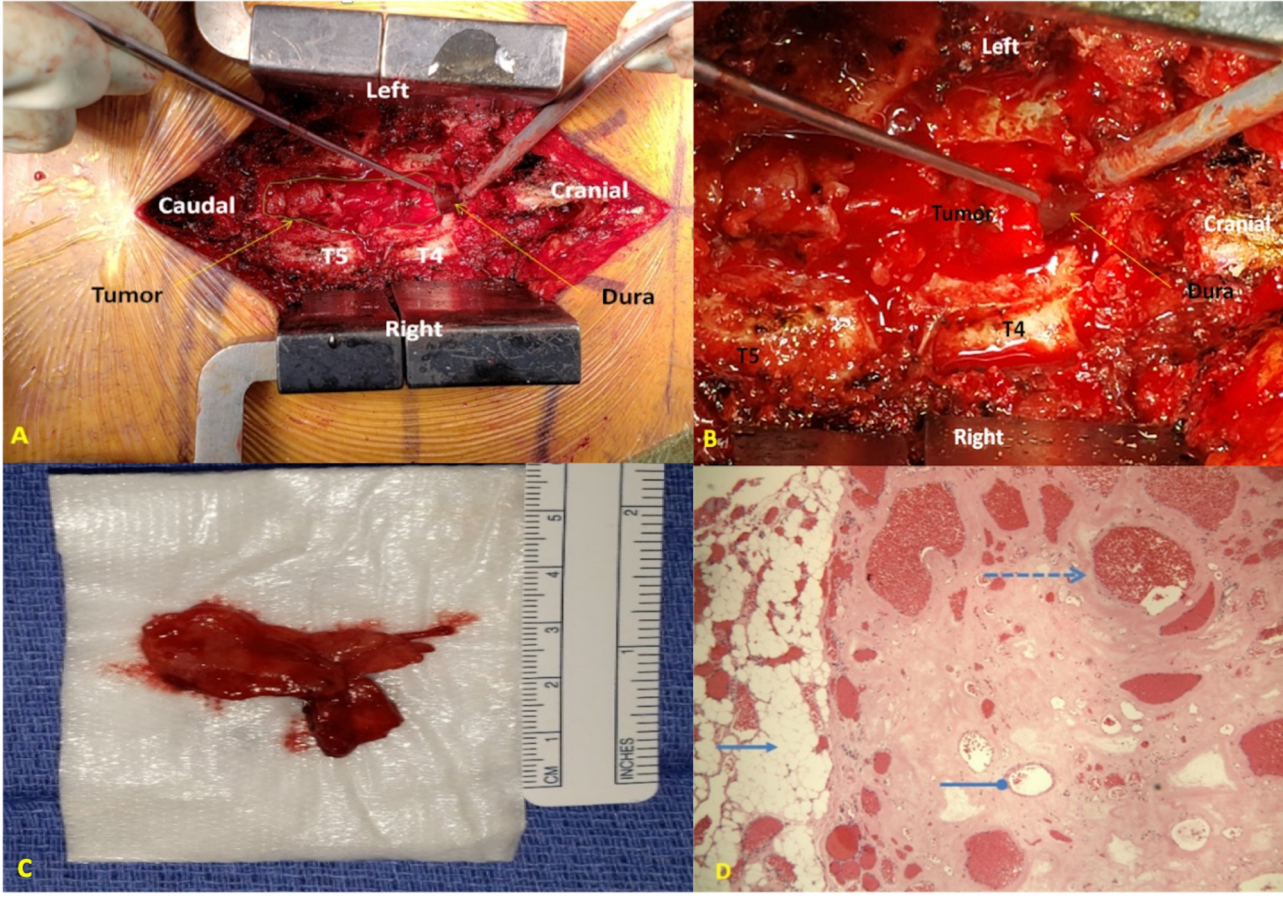
(A-C) The tumor is clearly epidural and was extremely vascular. The texture of the tumor was consistent with fat and was approximately 4 cm in length. (D) The pathology slides demonstrate adipose tissue (solid arrow on left), blood vessels (round head arrow), and microthrombi (dashed arrow).

**Figure 5:**
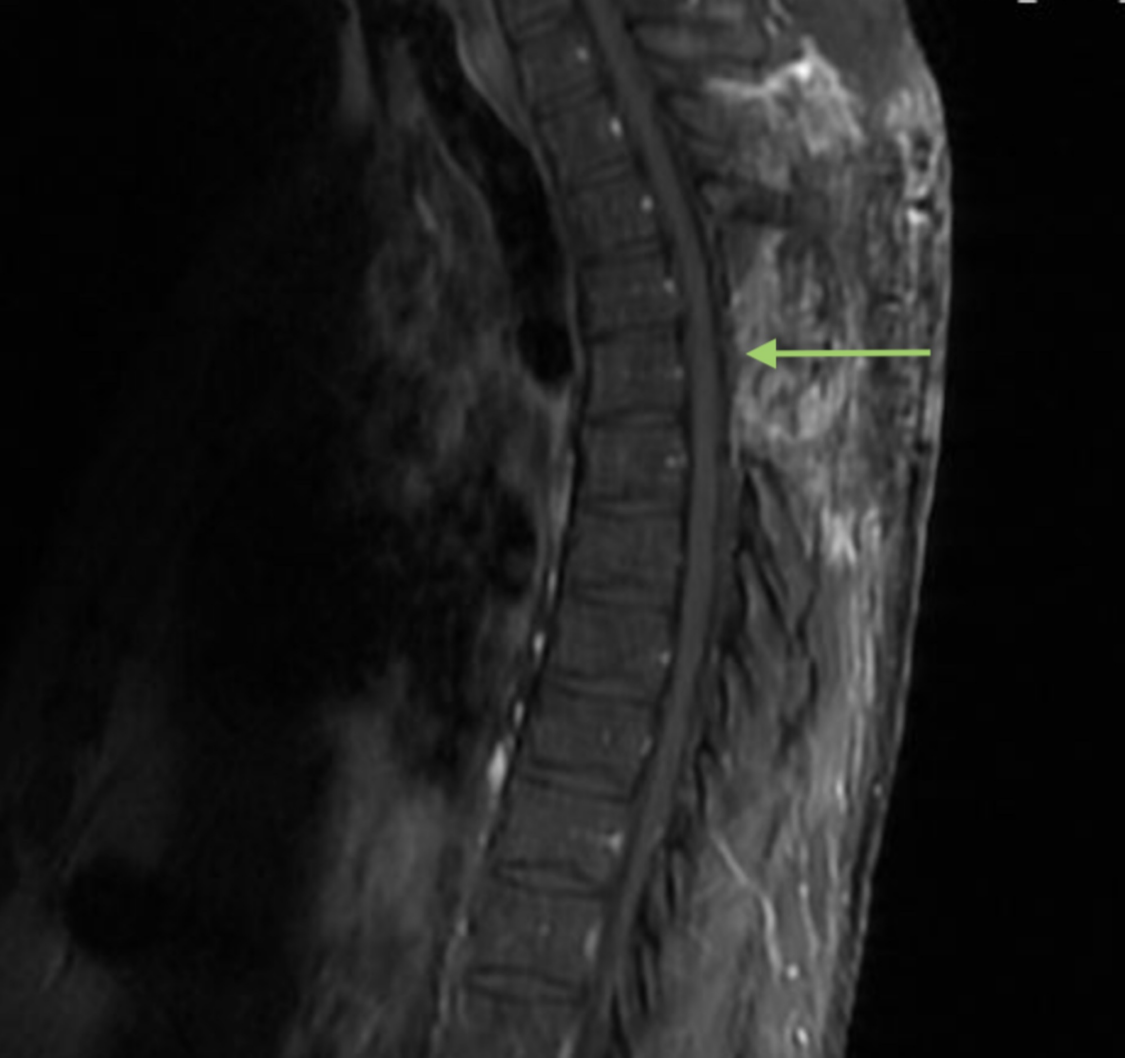
Post-operative MRI thoracic spine sagittal T1 fat suppression with contrast confirms gross total resection as indicated by the arrow.

The patient underwent a thoracic four and five laminectomy for resection of the mass. Intraoperative images are shown in Figure 4A and Figure 4B. Intraoperative pathology was read as angiolipoma with final pathology slides shown in Figure 4D. Surgery carried on until complete resection was achieved. Post-operatively the patient was extubated and recovered well with improved lower extremity paresthesias and transient urinary retention that resolved by post-operative day 2. Post-operative MRI demonstrated gross total resection as shown in Figure 5.

## Discussion

This case of angiolipoma diagnosis could have been missed or delayed since the patient’s symptoms did not clearly localize to the thoracic spine. Furthermore, there was no comment from the radiologist on the MRI scout image. Scout images are used for localization, but the scout images are not always interpreted by radiologists. In a study by Johnson et al., CT scout images that were retrospectively reviewed identified missed pathologic findings in 0.3-1% of cases [2]. Similarly, abnormalities were found in another 0.1-0.7% of CT cases but could not be confirmed retrospectively. In the same study, Johnson et al. quoted an estimated 85 million CT scans performed in 2011 and conservatively if 0.5% of abnormalities were missed, that could translate to 425,000 missed lesions [2]. While the previous study references CT imaging, this case demonstrates the value of evaluating MRI scout images. The clinical suspicion and evaluation of MRI scout imaging led to a dedicated MRI thoracic spine with and without contrast and subsequently to surgery.

In this case the final pathology was angiolipoma. While considered a benign lesion, neurologic decline can occur with hemorrhage, intratumoral thrombosis, or steal phenomenon [4-5]. Recurrence is rare, with only one case reported in literature over 12 years of surgical resection [6]. While angiolipoma is relatively benign, without tissue diagnosis this could have been a different type of tumor and clinical outcomes could have differed significantly. If these clinical outcomes lead to morbidity or mortality, could the surgeon be held liable? In order to answer this, looking at prior cases of lawsuits can be informative.

In one case, a pediatric patient was discharged from the ED after a CT head was read as normal. The patient later re-presented to the ED with change in mental status. A repeat CT head demonstrated an epidural hematoma, which ultimately resulted in death despite surgical intervention [7]. In this case the ED physician and radiologist were both named in a lawsuit for not identifying the initial skull fracture identified on scout imaging that is often associated with epidural hematomas and discharging the patient. A settlement was later reached in which both the radiologist's insurance company and ED physician's hospital paid in total $2.25 million [7].

In another case, a 16-year-old male involved in a motor vehicle rollover was initially ambulatory on scene and was found to have a T12-L1 flexion dislocation fracture identifiable on scout imaging. However, this was not seen on axial or reconstructions since the CT only included cervical, thoracic, and abdominal regions [8]. Consequently, the general surgeon discontinued spinal precautions, taking the patient to the operating room to repair a ruptured duodenum and the patient awoke with paraplegia. This case resulted in a settlement for $4.2 million shared by the surgeon, radiologist, and hospital [8].

As stated before there is no current standard or guideline mandating or setting interpretation of scout images as a standard of care [2,9-10]. In some training programs evaluating CT scout image for diagnosis is not customary [11]. There are likely programs that do emphasize reviewing the scout images. However, as cited that is not a uniform practice. Regardless of a physician’s training, plaintiff attorneys often point out that the fee for reading a scout image is the same whether it is performed or not. In addition, the radiation exposure is also the same whether read or not, so what excuse is there for not reading the scout images [10]? Any answer will likely be inadequate to a jury listening to the story of a grieving family or suffering patient, if that hardship could have been avoided with review of the scout images.

Returning to the case at hand, the scout images were for an MRI. If the MRI scout was not evaluated by the surgeon, would the surgeon be liable for any injury? To be held liable, four elements must be proven: professional duty to patient, breach of duty, injury caused by breach, and damages from injury [12]. A professional duty to the patient is typically interpreted as the establishment of the doctor-patient relationship. The point at which a neurosurgeon is consulted and begins to evaluate the patient, imaging, and provide recommendations, indicates that a relationship has been established. This can include covering patients for a colleague [12]. The standard of care defines a physician’s duty, therefore to prove a breach of duty expert testimony is often enlisted to provide medical insight to jury members who are often not from a medical environment. The breach of care must be the cause of patient injury. Finally, damages from the injury must be calculated and incorporate medical care of the injury, pain, and suffering. When these elements have been fulfilled, a lawsuit can be successfully brought upon a physician. Therefore, a treating neurosurgeon who does not review MRI scout images that later results in injury can be held culpable.

Interpretation of scout images can be difficult, especially in regards to MRI because the quality is not always optimal as the images are primarily used for localization. Suboptimal scout images could expose radiologists to liability with an expectation to identify lesions that might be anywhere whether in region of interest or not. Also, if a radiologist were to put a general blanket statement of clinical correlation needed for scout images, then this could expose the patient to unnecessary tests. It would be an improper use of resources if physicians begin ordering imaging of entire regions to protect themselves from missing lesions that might be on a scout that are not necessarily in the region of interest. It is the opinion of this team that scout images should not be included in permanent record for review. The images are obtained for technician use and should therefore only be used by technician at time of acquisition. Mandating that scout images be reviewed by physicians when not obtained for diagnostic purposes is unfair to patients and physicians. Expecting a diagnosis on images that are intentionally obtained with less quality when they can be clearer is unfair to the patient since the technology for the clearer picture is on the same machine. Similarly, expecting physicians to identify incidental findings when lesions are not in the area of high-quality diagnostic images can lead to missed diagnoses. As always if there is a clinical concern, then dedicated images can be obtained of the area of interest for diagnostic purposes. Currently with no clear guidelines, patients are at risk of lesions being missed on scout imaging and physicians are at risk of hurting patients.

## Conclusions

Lesions that are not addressed on scout imaging can lead to missed diagnoses. Review of the scout images holds value in providing a gestalt, but also in sometimes identifying areas of concern that radiologist might not address since the surgeon has the advantage of seeing and examining the patient. However, expecting physicians to evaluate suboptimal images exposes physicians to the risk of hurting patients. Further research is needed in the potential implications of mandating review of scout images and/or elimination of scout images from medical record.
